# Isolation of a Psychrotolerant and UV-C-Resistant Bacterium from Elephant Island, Antarctica with a Highly Thermoactive and Thermostable Catalase

**DOI:** 10.3390/microorganisms8010095

**Published:** 2020-01-10

**Authors:** María T. Monsalves, Gabriela P. Ollivet-Besson, Maximiliano J. Amenabar, Jenny M. Blamey

**Affiliations:** 1 Fundación Científica y Cultural Biociencia, José Domingo Cañas 2280, Ñuñoa, Santiago 7750132, Chile; mtmonsalves@bioscience.cl (M.T.M.); gollivet-besson@bioscience.cl (G.P.O.-B.); amenabar.barriuso@gmail.com (M.J.A.); 2 Facultad de Química y Biología, Universidad de Santiago de Chile, Avenida Libertador Bernardo O’Higgins 3363, Estación Central, Santiago 9170022, Chile

**Keywords:** Antarctica, *Serratia*, psychrotolerant, UV-C radiation, catalase, thermostable, antioxidant, ROS, oxidative stress

## Abstract

Microorganisms present in Antarctica have to deal not only with cold temperatures but also with other environmental conditions, such as high UV radiation, that trigger the generation of reactive oxygen species. Therefore, Antarctic microorganisms must have an important antioxidant defense system to prevent oxidative damage. One of these defenses are antioxidant enzymes, such as catalase, which is involved in the detoxification of hydrogen peroxide produced under oxidative conditions. Here, we reported the isolation and partial characterization of an Antarctic bacterium belonging to the *Serratia* genus that was resistant to UV-C radiation and well-adapted to cold temperatures. This microorganism, denominated strain I1P, was efficient at decreasing reactive oxygen species levels produced after UV-C irradiation. Genomic and activity assays suggested that the enzymatic antioxidant defense mechanisms of strain I1P, especially its catalase enzyme, may confer UV resistance. This catalase was active in a wide range of temperatures (20–70 °C), showing optimal activity at 50 °C (at pH 7.0), a remarkable finding considering its psychrotolerant origin. In addition, this enzyme was thermostable, retaining around 60% of its activity after 6 h of incubation at 50 °C. The antioxidant defense systems of strain I1P, including its surprisingly thermoactive and thermostable catalase enzyme, make this microorganism a good source of biocompounds with potential biotechnological applications.

## 1. Introduction

Antarctica is one of the most extreme and pristine environments on Earth. Along with low temperatures and low water activities, high ultraviolet (UV) radiation is also present in this continent [1,2,3,4]. These environmental stresses trigger the generation of reactive oxygen species (ROS), including superoxide anions (O_2_^−^), hydrogen peroxide (H_2_O_2_), and the highly reactive hydroxyl radicals (^•^OH), which could lead to oxidative stress if the antioxidant mechanisms of the cell are overcome by pro-oxidant agents [3]. These species are highly reactive, causing oxidative damage and altering the structure and function of macromolecules, such as DNA, lipids, and proteins [5,6,7,8]. Therefore, in order to inhabit these environments, Antarctic microorganisms must have an important antioxidant defense system to prevent oxidative damage [1,9,10]. These defense systems are classified into enzymatic or non-enzymatic based on the nature of the antioxidant agents [11]. The non-enzymatic mechanism involves the use of glutathione, vitamins, and/or pigments to prevent oxidative damage [3,12]. Among these antioxidant agents, it has been shown that pigments might play a dual role by protecting cells not only against oxidative damage but also against osmotic shock [3,12]. Some examples of Antarctic microorganisms that use these non-enzymatic defense mechanisms include *Pedobacter* sp., a psychrololerant bacterium that produces different types of carotenoids with a strong antioxidant capacity, protecting cells against lipid peroxidation and ROS induced by UV radiation [3]. Similarly, *Flavobacterium* sp., *Arthrobacter* sp., and *Sphingomonas* sp., all of them isolated from Antarctica, also produce pigments that protect cells against UV radiation [13].

The enzymatic mechanism involves the use of enzymes, such as superoxide dismutase (SOD), catalase (CAT), and/or glutathione peroxidase (GPx), as the protecting agents against oxidative damage [11,14]. The mechanisms involved in the enzymatic antioxidant reaction are various and oftentimes work in synchrony against ROS. For example, SOD catalyzes the dismutation of O_2_^−^ into oxygen (O_2_) and H_2_O_2_ and then CAT degrades H_2_O_2_ into O_2_ and water [14,15,16]. Some examples of Antarctic microorganisms that rely on these enzymes against oxidative stress include *Pseudomonas* sp., *Bacillus* sp., and *Marinomonas* sp. [17]. Other Antarctic microorganisms, such as *Colwellia psychrerythraea*, rely on both non-enzymatic mechanisms, through the use of reduced glutathione, which can protect cells against free radicals, and enzymatic defense mechanisms, through the use of CAT and SOD [18].

Due to their properties, these antioxidant enzymes have also been of interest in biotechnology [16,19]. In particular, CAT has been widely used in several industrial applications, including food or textile processing, to remove H_2_O_2_ that is used for sterilization or bleaching purposes, respectively [20,21,22,23,24].

The growing demand for antioxidant enzymes in the market [16,20] in addition to the increasing levels of UV radiation at which organisms are exposed to, mainly due to ozone depletion in the atmosphere [2], have led to an increasing interest in ROS detoxification studies involving enzymatic defense mechanisms. To this end, Antarctica represents a great natural laboratory to study UV-resistant microorganisms that could reveal new antioxidant compounds or enzymes with novel properties.

In this work, we reported the isolation and partial characterization of a psychrotolerant and radio-resistant bacterium from Elephant Island, Antarctica. The ability of this microorganism to resist UV-C radiation prompted additional experiments aimed at further study of the mechanisms allowing these cells to deal with ROS generation. Especially, we aimed to determine if these cells were capable of decreasing ROS levels after UV-C radiation and to shed some light on the potential mechanisms involved. The results suggest that the enzymatic antioxidant defense mechanisms of this Antarctic bacterium, especially its CAT enzyme, may be important for conferring UV-C resistance. In addition, the enzymatic and stability properties of the purified CAT make this enzyme a good candidate for potential biotechnological applications.

## 2. Materials and Methods

### 2.1. Sample Collection and Enrichment of Strain I1P

Samples for enrichments and isolation were collected from Elephant Island (61°08′ S 55°07′ W), Antarctica, during the Antarctic Chilean Expedition 46 (ECA 46). Surface soil samples (~8 °C; pH ~6.5) were sampled aseptically using a flame-sterilized spatula, placed in sterile vials, and stored for transport to the laboratory where they were used for culture enrichments. Roughly ~500 mg of the environmental sample was used to inoculate an aerobic media containing tryptone (10 g L^−1^), yeast extract (5 g L^−1^), and NaCl (60 g L^−1^). The final pH of the medium was 7.0. In total, 100 mL of the medium were dispensed into 250-mL bottles and were subjected to autoclave sterilization. Following autoclave sterilization, the bottles were inoculated with the soil samples recovered from Elephant Island and were incubated at 8 °C for up to two weeks. The progress of culture enrichments was monitored by phase-contrast microscopy (Eclipse 80i, Nikon, Tokyo, Japan). Following 2 rounds of dilution to extinction in conjunction with colony isolation from solid media, a single morphotype was observed, which was designated as strain I1P.

### 2.2. DNA Extraction, 16S rRNA Gene Amplification, and Phylogenetic Analysis

Genomic DNA was extracted from the isolate using a modified phenol–chloroform extraction as previously described [25]. DNA extract was subjected to PCR amplification of archaeal and bacterial 16S rRNA genes. PCR amplification of the 16S rRNA gene was performed according to previously described protocols [26] using archaeal primers 21F (5’-TCCGGTTGATCCYGCCGG-3’) and 1492R (5’-GGTTACCTTGTTACGACTT-3’) and bacterial primers 334F (5’-CCAGACTCCTACGGGAGGCAGC-3’) and 939R (5’-CTTGTGCGGGCCCCCGTCAATTC-3’). Archaeal 16S rRNA gene amplicons were not detected. Bacterial 16S rRNA gene amplicons were purified, sequenced, assembled, and analyzed using previously published methods [27]. Briefly, PCR products were purified with the commercial kit, Wizard PCR Preps DNA Purification System (Promega, Madison, WI, USA), following the instructions provided by the manufacturer. Purified products were sequenced using an ABI 3730xl automated DNA sequencer (Applied Biosystems, Foster city, CA, USA) on the Biomedical Core Research from the University of Michigan. Sequences were assembled using the BioEdit sequence alignment editor freeware (version 7.2.5) (Ibis Therapeutics, Carlsbad, CA, USA) [28] and obtained contigs were subjected to nucleotide–nucleotide Basic Local Alignment Search Tool (BLASTn) analysis [29] against the “nr” database provided by the National Center for Biotechnology Information (NCBI). ClustalW software (University College Dublin, Dublin, Ireland) was used to align the partial sequence of the 16S rRNA gene with those of the type strains of species of the genus *Serratia* retrieved from GenBank. The software package MEGA6 (Pennsylvania State University, PA, USA) [30] was used for phylogenetic analysis using the neighbor-joining method [31]. Distances were computed using the maximum composite likelihood method [32] with a bootstrap analysis of 1000. Nucleotide sequences of the 16S rRNA gene of strain I1P were deposited in the GenBank database under the accession number MN011068.

### 2.3. Morphological, Physiological and Biochemical Characterizations

Cell morphology was examined by phase-contrast microscopy (Eclipse 80i, Nikon, Tokyo, Japan). The temperature range for the growth of strain I1P was tested between 4 to 40 °C, at pH 7.0 (optimal pH). The pH range for growth was tested between 4.0 and 11.0, at 22 °C (optimal temperature). The salinity range for growth was tested between 1% and 21% NaCl at pH 7.0 and 22 °C. Biochemical characterization was performed using the API 20 E Kit (bioMérieux, Inc., Marcy- l’Étoile, France). Gram stain was determined using the Difco Gram-staining kit (BD Difco™ BBL™, BD, Drogheda, United Kingdom).

### 2.4. Effect of UV Radiation on Cell Viability

UV radiation tolerance was studied by exposing sterile Petri plates containing 5 mL of liquid culture (OD600 = 0.4) to UV-C radiation using previously described protocols [9]. A specially designed dark chamber equipped with a UV-C lamp was used to irradiate cultures. Briefly, cultures were placed 30 cm away from the UV-C lamp and exposed to UV-C radiation for 2 h. Then, 100-μL aliquots were taken at different time intervals, inoculated in Petri plates with solid LB medium, and incubated in optimal conditions (see below) for 24 h. Cell viability was determined by colony forming units (CFUs) per mL and expressed as the percent of viable cells. *Escherichia coli* strain BL21 (Promega, Madison, WI, USA) and *Geobacillus* sp. strain GWE1 (personal culture collection) [33] were used as control microorganisms. *E. coli* strain BL21, strain GWE1, and strain I1P were grown in Luria- Bertani (LB) medium (at 37 °C), LB/3 medium (at 70 °C), and LB 6% NaCl (at 22 °C), respectively. The irradiance of the UV-C lamp was quantified with a radiometer (VLX-3W; Vilber Lourmat, Marne-la-Vallée, France) equipped with a UV-C sensor. The UV-C sensor was placed inside a dark chamber at the same distance the cultures were placed. The average intensity of the lamp in addition with the UV-C radiation dose (intensity × time) was determined by the radiometer.

### 2.5. Detection of Reactive Oxygen Species (ROS)

For the quantification of ROS species, a free radical probe 2’,7’-dichlorodihydrofluoresceindiacetate (H_2_DCFDA) (Sigma, San Louis, MO, USA) was used as previously described [3]. Cells of *E. coli*, strain GWE1, and strain I1P were grown using the conditions described in the previous section. Cultures were grown to OD600 = 0.4 and immediately exposed to UV-C radiation for 4 min. Then, cells were washed with 10 mM potassium phosphate buffer (pH 7.0), incubated for 30 min in the same buffer containing 10 µM H_2_DCFDA (dissolved in dimethyl sulfoxide), and washed and resuspended in potassium phosphate buffer (10 mM, pH 7.0). Finally, cells were disrupted by sonication, cell debris was removed by centrifugation (10 min 13,000× *g*), and the fluorescent intensity was measured in a fluorescence multi-well plate reader (Biotek series FLX 800 TBI, Biotek, Bad Friedrichshall, Germany) (excitation, 490 nm; emission, 519 nm). Results were normalized per mg of protein and expressed as the percent of fluorescence for each strain. The fluorescence intensity of the unexposed control (time 0) was assigned 100% of the fluorescence and this value used to compare the fluorescence intensity at the different time points. A negative and positive control for each strain were used to detect the baseline of the fluorescence intensity and determine the concentration of the probe to obtain an optimal signal, respectively. The negative control corresponded to cells treated with the probe but without any other treatment that could induce ROS (i.e., UVC radiation). The positive control corresponded to cells treated with the prove and an elicitor of ROS (UVC radiation for 5 min).

### 2.6. Enzyme Assay

Catalase activity was measured spectrophotometrically by monitoring the decrease in absorbance at 240 nm due to the transformation of H_2_O_2_ to H_2_O and O_2_. The reaction mixture contained 10 mM H_2_O_2_ and 50 mM potassium phosphate (pH 7.0). One unit (U) of CAT activity was defined as the decomposition of 1 µmol of H_2_O_2_ per minute [34]. Peroxidase activity was monitored at 420 nm with pyrogallol as the substrate [35]. SOD activity was assayed based on the ability of SOD to inhibit the reduction of nitroblue tetrazolium (NBT) as previously described [14]. One U of SOD activity was defined as the amount of enzyme that inhibited the reduction of NBT by 50%. The protein concentration was measured by the method of Bradford [36] using a commercial assay kit (Bio-Rad, Berkeley, CA, USA).

### 2.7. Effect of UV Radiation on Enzyme Activity

The effect of UV-C radiation on SOD and CAT activities was studied by exposing sterile Petri plates containing 5 mL of liquid culture (OD600 = 0.4) to UV-C radiation using previously described protocols [9]. Cultures were exposed to UV-C radiation for 50 min and aliquots were taken at different time intervals and assayed for SOD and CAT activities. Enzyme activities at each time point were compared to the enzyme activities of the unexposed cells (time 0), which were assigned 100% of the relative activity. Measurements were performed in triplicate.

### 2.8. Enzyme Purification

Catalase enzyme was routinely purified from I1P cells at 22 °C. For the preparation of the crude extract, 20 g of cells were lysed with a modified cellular disruption method [37]. Briefly, cells were resuspended in 80 mL of 50 mM Tris HCl buffer (pH 7.5) containing 1 mM EDTA and lysozyme (1 mg·mL^−1^) and incubated at 37 °C for 1 h. Then, the sample was disrupted using a sonicator (Branson sonifier 450, Branson, Danbury, CT, USA) and the cell debris was removed by centrifugation (9000× *g* for 30 min). The final supernatant was then used as the crude extract for the purification step. The crude extract was loaded onto a column (XK 16/20, GE Healthcare, Chicago, IL, USA) of DEAE-Sepharose Fast Flow (Pharmacia Biotech, Stockholm, Sweden) previously equilibrated with 50 mM Tris HCl buffer (pH 8.0). The enzyme was eluted with a linear gradient of NaCl 0 to 1 M in Tris HCl buffer (pH 8.0) with a flow rate 1 mL·min^−1^. Fractions containing CAT activity were combined, concentrated to a volume of 1 mL by ultrafiltration (PM-30 membrane filter; Amicon, Burlington, MA, USA), and applied to a column (Tricorn 10/600, GE Healthcare, Chicago, IL, USA) of Superdex-200 (Pharmacia Biotech, Stockholm, Sweden) previously equilibrated with 0.2 M NaCl in 50 mM Tris HCl buffer (pH 8.0). Fractions with CAT activity were then loaded to a column Q-HiTrap Fast Flow (Pharmacia Biotech, Stockholm, Sweden) previously equilibrated with 50 mM Tris HCl buffer (pH 8.0). The enzyme was eluted with a linear gradient of NaCl 0 to 1 M in Tris HCl buffer (pH 8.0) with a flow rate of 1 mL·min^−1^. All columns were controlled by a Pharmacia FPLC system (Pharmacia Biotech, Stockholm, Sweden).

### 2.9. Molecular Mass Determination

The apparent molecular mass of the native CAT was estimated by gel filtration chromatography on a Superdex-200 column (Tricorn 10/600, GE Healthcare, Chicago, IL, USA) (Pharmacia Biotech, Stockholm, Sweden) as previously described [14]. Briefly, a Superdex-200 column was equilibrated with 50 mM Tris HCl buffer (pH 8.0) containing 0.2 M NaCl and calibrated using bovine GDH (300.0 kDa), BSA (66.0 kDa), ovalbumin (45.0 kDa), and lysozyme (14.3 kDa) as the standard proteins. Blue dextran was used to determine the void volume. The subunit molecular mass was determined by sodium dodecyl sulfate-polyacrylamide gel electrophoresis (SDS-PAGE) (12%) according to the method of Laemmli [38] using a wide range marker (Thermo Scientific, Waltham, MA, USA) with the following proteins: β-galactosidase (120 kDa), BSA (85 kDa), ovalbumin (50.0 kDa), cardonic anhydrase (35 kDa), β-lactoglobulin (25 kDa), and lysozyme (20 kDa). Gel was stained with 0.2% Coomassie Brillian Blue R250.

### 2.10. Protein Identification

Catalase was identified by Matrix-Assisted Laser Desorption/Ionization – Time-Of-Flight (MALDI TOF/TOF) in the National Center for Biotechnology, Spain. Briefly, protein samples were digested with trypsin and analyzed with a MALDI TOF/TOF mass spectrometer. Mass spectra (*m*/*z*) corresponding of the tryptic peptides were obtained and analyzed against the National Center for Biotechnology Information Non-redundant (NCBInr) database.

### 2.11. Effects of pH and Temperature on Catalase Activity

The pH dependence of CAT activity was determined between pH 5.5 and 8.0 using the following buffers: 50 mM MES (pH 5.5–6.5), 50 mM phosphate (pH 7.0), 50 mM EPPS (pH 7.5), and 50 mM Tris HCl (pH 8.0). The temperature range of CAT activity was determined between 20 and 70 °C, at pH 7.0.

### 2.12. Thermostability

For determination of CAT thermostability, the enzyme was placed in small tubes with O-ring-sealed caps and incubated at 50 °C in a dry bath (MD-02N-220, Major Science, Saratoga, CA, USA) for 0 to 6 h, as previously described [37]. Samples were taken at different times and assayed for enzyme activity. Residual activity was determined at 50 °C.

### 2.13. Effect of Inhibitors on Catalase Activity

Catalase activity was tested in the presence of different concentrations of inhibitors, including 3-amino-1,2,4-triazole (75 mM) [39], potassium cyanide (10 mM) [39], and H_2_O_2_ (80 mM) [39,40]. The reaction mixture was incubated for 2 min at 25 °C, and the remaining activity was quantified according to Allgood and Perry [39]. The effect of organic solvents on CAT activity was also evaluated. The enzyme was mixed with ethanol and chloroform (10:5:3) and vortexed for 10 min at room temperature [41]. Then, CAT activity was monitored spectrophotometrically at 240 nm as previously described.

### 2.14. Statistical Analyses

Two-tailed Student’s t-tests were used to evaluate the statistical significance (*p* < 0.05) of differences between datasets and were conducted in Microsoft Excel.

## 3. Results and Discussion

Antarctica could be considered as one of the wildest, extreme, and more isolated continents on Earth. Microbes in Antarctica have to deal with different extreme conditions, including low temperatures, low water activities, high salt concentrations, and/or high UV radiation [1,4]. These conditions often occur in synchrony, selecting for microorganisms that can withstand different extreme conditions simultaneously [1,9,10]. To this end, two of the most ubiquitous conditions to which microorganisms are exposed to in Antarctica are cold temperatures and high UV radiation [2,3]. Since these environmental stresses trigger the generation of ROS [3,42], microorganisms inhabiting these environments must have an important antioxidant defense system to prevent oxidative damage.

To study if microorganisms present in Antarctica are adapted to cold temperatures and UV radiation, and to shed some light on the mechanisms allowing these cells to deal with ROS generation, we isolated and partially characterized a psychrotolerant and radio-resistant microorganism from Elephant Island, Antarctica, designated as strain I1P. This rod-shaped microorganism was gram negative (Supplementary Figure S1) and grew in a temperature range from 4 to 37 °C (optimum growth at 22 °C) and in a pH range from 5 to 10 (optimum growth between 7 and 9). The salinity range for growth of strain I1P was 2% to 19% NaCl (optimum growth between 2% and 6%). Its biochemical characterization using the API 20 E Kit (Table 1) indicated that strain I1P was positive for L-tryptophane deaminase, gelatinase, reduction of nitrates to nitrites (NO_2_), and reduction of nitrates to nitrogen gas (N_2_). Negative results were obtained for β-galactosidase, arginine dihydrolase, lysine decarboxylase, ornithine decarboxylase, citrate utilization, H_2_S production, urease, indol production, and acetoin production. All fermentation/oxidation tests for carbohydrates were negative in the API20 E strips.

**Figure 1:**
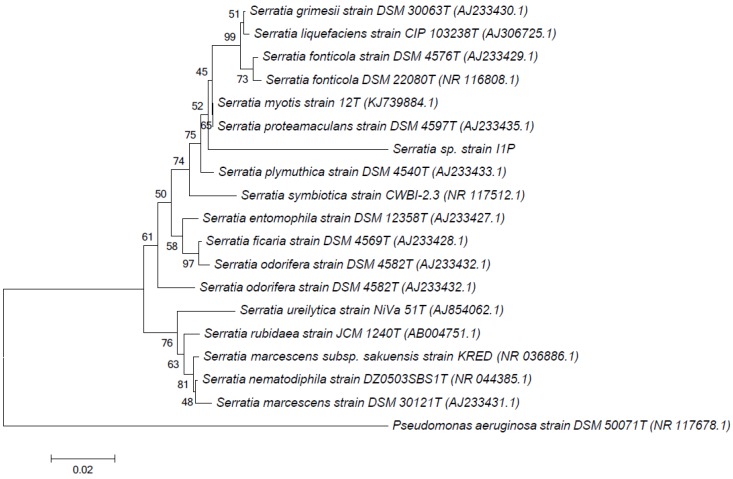
Neighbor-joining phylogenetic tree based on 16S rRNA gene sequences of species from the *Serratia* genus. The percentage of replicate trees in which the associated taxa clustered together in the bootstrap test (1000 replicates) are shown next to the branches. *Pseudomonas aeruginosa* DSM 50071T was used as an out-group. Bar, 2 nucleotide substitutions per 100 nucleotides.

Analysis of the 16S rRNA gene partial sequence indicated that strain I1P was 96.41% identical to the 16S rRNA gene from *Serratia proteamaculans* (closest cultured microorganism). This 16S rRNA gene sequence similarity was below the species delineation threshold value of 98.7% suggested by Stackebrandt and Ebers [43] or <97% as suggested by other authors as the threshold for a new specie [44]. The evolutionary history of the 16S rRNA gene sequence from strain I1P, inferred using the neighbor-joining method (Figure 1), shows the phylogenetic placement of strain 1IP within the *Serratia* genus. Although *S. proteamaculans* was the closest cultured microorganism, strain 1IP formed a separate branch from this specie. These differences between 16S rRNA gene sequences, in addition to the biochemical differences between strain I1P and *S. proteomaculans* [45,46], particularly the l-tryptophane deaminase activity present in I1P but not in other strains from *S. proteomaculans* [45,46] and the absence of β-galactosidase, lysine decarboxylase, and ornithine decarboxylase activities and the inability to use citrate or ferment/oxidize carbohydrates by I1P in comparison to other strains from *S. proteomaculans* [45,46], suggests that I1P may represent a novel specie within the *Serratia* genus. However, more information is required to further support this observation.

Consistent with members of the *Serratia* genus [47], strain I1P was gelatin and nitrate positive, displayed salt tolerance, and grew at low temperatures and alkaline pH. Although *Serratia* strains have been previously isolated from cold temperature environments, including Antarctica [48,49,50], to our knowledge, there is no report about the effect of UV-C radiation on cell viability in members from this genus. To evaluate the effect of UV-C radiation in strain I1P, the cell viability of UV-C-exposed cultures was studied. *E. coli* and *Geobacillus* sp. strain GWE1 were used as “control cultures” for UV-sensitive and UV-resistant microorganisms, respectively [14]. As expected, *E. coli* was not resistant to UV-C radiation, being completely unviable after 5 min of irradiation (Table 2). In comparison, *Geobacillus* sp. strain GWE1 was more resistant to UV-C radiation, likely due to its efficient enzymatic defense mechanism against ROS [14]. Strain I1P was remarkably more resistant to UV-C radiation than both control microorganisms, being able to form colonies even after 120 min of UV-C irradiation (final UV-C dose 6.33 Jls/cm^2^) (Table 1). Although *Geobacillus* sp. strain GWE1 and *Serratia* sp. strain I1P were able to form colonies after 120 min of UV-C irradiation, strain GWE1 lost over 90% viability after 5 min of UV-C irradiation. Differences in the cell viability between strains were statistically significant (*p* < 0.05). These results suggest that strain I1P must have an efficient antioxidant defense mechanism that allows these cells to deal with ROS generation to prevent oxidative damage, particularly during the first stages of UV exposure [11,51,52,53,54].

**Figure 2:**
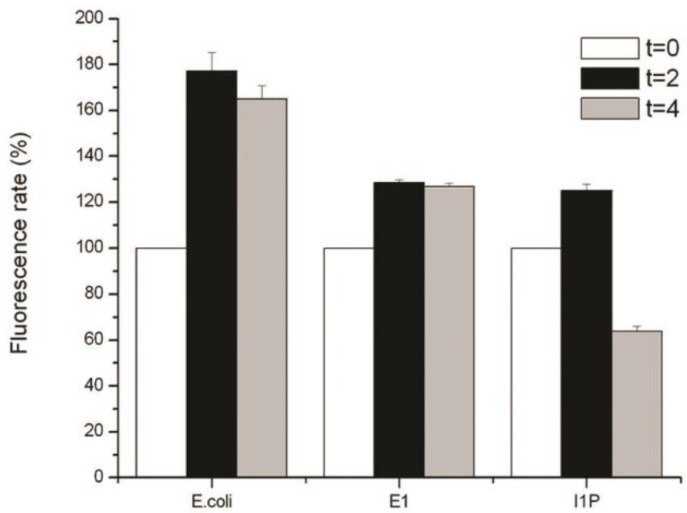
Intracellular levels of reactive oxygen species (ROS) measured with the oxidant-sensing probe 2’,7’-dichlorodihydrofluorescein diacetate. Cells were exposed to UV-C radiation for 2 and 4 min and their fluorescence intensity compared to the unexposed control (time 0), which was assigned 100% of the fluorescence. Measurements were performed in quintuplicate.

In order to study the antioxidant response to UV radiation, intracellular ROS levels were measured with the free radical probe H_2_DCFDA. This probe is sensitive to peroxynitrite anion, peroxyl radical, and H_2_O_2_. All these ROS can cause oxidative stress if the antioxidant defense system is overcome. Cultures of *E. coli*, *Geobacillus* sp. strain GWE1, and *Serratia* sp. strain I1P were exposed to UV-C radiation for 2 and 4 min and their fluorescence intensity compared to the unexposed cells (time 0), which were assigned 100% of the fluorescence. Cultures were only irradiated for 4 min, since *E. coli* was not viable after 5 min of irradiation with UV-C (Table 1), preventing a comparison of ROS production over this time. Although the fluorescence intensity could be directly measured on intact cells, cell lysis considerably increased the fluorescent signal, facilitating the measurements. Because of this, and also based on previous reports that disrupted cells for ROS determination when using this free radical probe, we lysed the cells before the fluorescent measurements. All cultures showed a statistically significant (*p* < 0.05) increase in intracellular ROS production after UV-C radiation, with *E. coli* being the microorganism that produced more ROS after UV-C treatment (~80% after 2 min) (Figure 2). Although both *Geobacillus* sp. strain GWE1 and *Serratia* sp. strain I1P displayed a ~30% increase in ROS levels after 2 min of UV-C exposure, strain I1P was more efficient at decreasing these ROS levels after 4 min of irradiation (Figure 2). This suggested that *Serratia* sp. strain I1P must have an efficient antioxidant defense system against UV radiation, in comparison to *E. coli* and *Geobacillus* sp. strain GWE1.

**Figure 3:**
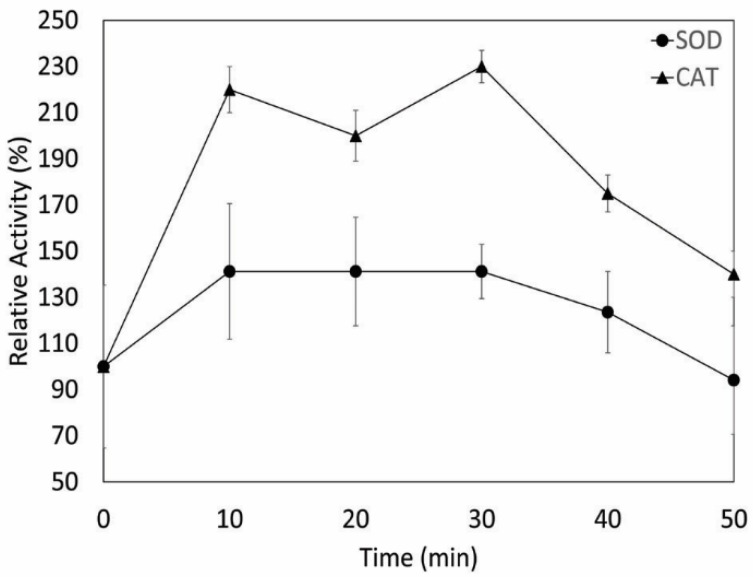
Effect of UV-C radiation on superoxide dismutase (SOD) and catalase (CAT) activities of I1P. Cells were exposed to UV-C radiation for 50 min. SOD and CAT activities were measured at different lengths of time and compared to the enzyme activities of the unexposed cells (time 0), which were assigned 100% of the relative activity. Measurements were performed in triplicate.

To date, two main defense mechanisms against oxidative damage have been described [11]. One is non-enzymatic, involving the use of glutathione, vitamins, and pigments, such as carotenoids, to prevent oxidative damage [3,12]; and the second is an enzymatic mechanism involving the use of enzymes, such as SOD, CAT, and/or GPx [11,14]. Genomic analysis of *Serratia* sp. strain I1P (protected data, unavailable) suggested a potential role for both non-enzymatic (through glutathione) and/or enzymatic defense mechanisms (through SOD, CAT, or GPx) against oxidative stress. Preliminary enzyme activity screening on crude extracts from strain I1P revealed both SOD (1.37 U/mg) and CAT (25 U/mg) activities, consistent with the ability of this microorganism to withstand UV-C radiation. To further evaluate the role of these antioxidant enzymes in the resistance of I1P against UV-C radiation, SOD and CAT activities were measured in cells exposed to UV-C radiation. SOD and CAT activities increased approximately 1.4- and 2.2-fold, respectively, after 10 min of UV-C radiation, in comparison to the unexposed cells (time 0) (Figure 3). The bigger increase in CAT activity after UV-C radiation, in addition to the higher specific activity (~18-fold) of CAT related to SOD, suggests that CAT contributes in a higher degree to the antioxidant response of I1P cells than SOD. The role of CAT conferring UV-C radiation resistance was further supported by the use of a recombinant *E. coli* BL21 strain expressing the gene that encodes the CAT enzyme from I1P (Monsalves et al. in prep). This recombinant strain was irradiated with UV-C radiation for 35 min and the cell viability was assayed at different intervals of time. In comparison to *E. coli* strain BL21 without the plasmid carrying the gene that codes for CAT (negative control), the recombinant strain was more resistant to UV-C radiation, highlighting the role of this enzyme in conferring UV-C radiation (Supplementary Table S1).

**Figure 4:**
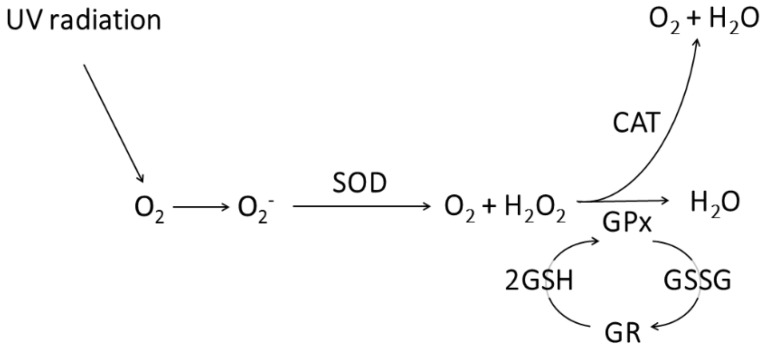
Proposed antioxidant defense mechanism of strain I1P. SOD: superoxide dismutase; CAT: catalase; GPx: glutathione peroxidase; GR: glutathione reductase; GSH: reduced glutathione; GSSG: glutathione disulfide.

Based on genomic data and enzymatic activity measurements of I1P, it is likely that a set of different antioxidant defense mechanisms are simultaneously involved in the resistance to UV-C radiation. To this end, O_2_^−^ produced in UV-C-exposed cells is degraded into O_2_ and H_2_O_2_ through the activity of SOD and then CAT degrades H_2_O_2_ into O_2_ and water [14,15,16]. Additionally, GPx could also reduce H_2_O_2_ to H_2_O by oxidizing two molecules of reduced glutathione to glutathione disulfide, and then the enzyme glutathione reductase, which is also encoded in the I1P genome, could reduce the oxidized glutathione to complete the cycle [55,56] (Figure 4).

Among these antioxidant agents, SOD and CAT have been of great interest in biotechnology in the last decades [19]. The high activity of CAT in comparison to the other antioxidant enzymes from I1P prompted further analyses to purify and characterize this enzyme in order to address the potential use of this CAT in biotechnological applications. Table 3 shows the results of the purification method developed for the CAT from strain I1P. An increment in the specific activity and a decrease in the amount of protein with each purification step was observed. The enzyme was purified 298-fold compared to the crude extract, with a 21% recovery of activity after three successive chromatographic steps. The apparent molecular mass of the native enzyme was 223.8 kDa as estimated by size exclusion chromatography using a Superdex-200 column. Based on SDS-PAGE gel electrophoresis, the purified enzyme was found to be composed of subunits of an estimated molecular mass of 62.7 kDa (Supplementary Figure S2). The experimental results obtained from the size exclusion chromatography and gel electrophoresis suggest that the native enzyme has a tetrameric structure, composed of four identical or similar subunits. In order to confirm the identity of the CAT enzyme, the 62.7-kDa band was excised from SDS-PAGE and subjected to MALDI TOF/TOF analysis. The experimentally obtained masses were compared with the theoretical peptide masses of proteins stored in the NCBInr database using the mass search program Mascot as previously described [37]. The result of the peptide mass fingerprinting showed that the enzyme matched with the information reported for the CAT from *Serratia proteamaculans* 568.

**Figure 5:**
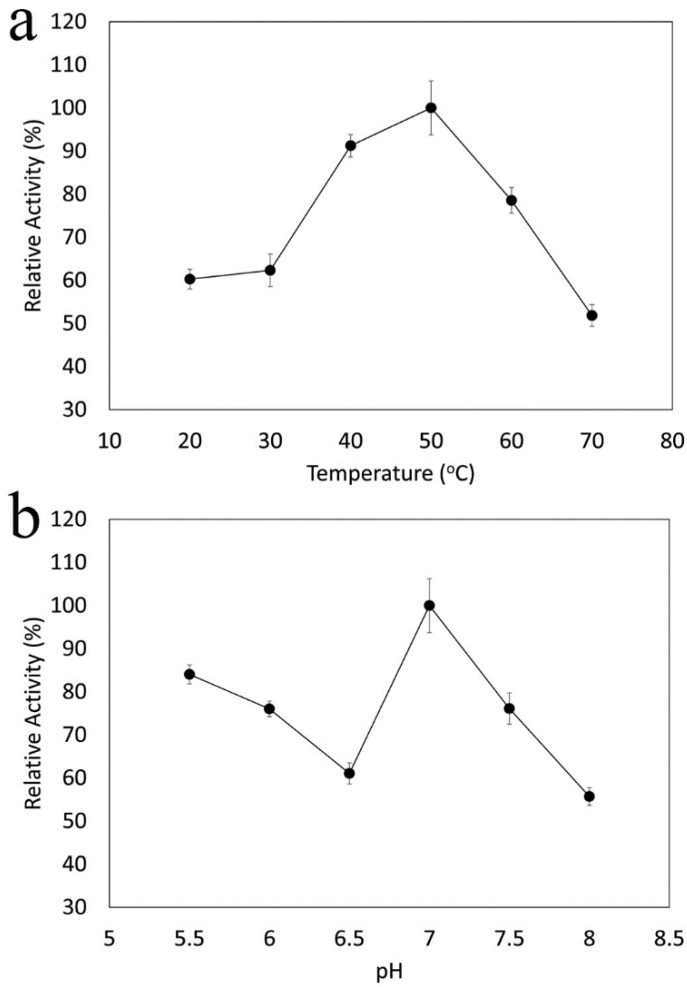
Optimal temperature (**a**) and pH (**b**) of I1P catalase activity. Temperature and pH range of catalase activity was determined at pH 7.0 and 50 °C respectively. Here, 100% of catalase activity represents the maximum activity measured. Measurements were performed in triplicate.

To evaluate the potential role of this CAT enzyme in biotechnological applications (i.e., biocatalysts must be stable and active in the harsh conditions at which industrial processes usually take place), the optimal pH and temperature for the CAT activity in addition to stability studies were performed. The effects of temperature and pH on CAT activity are shown in Figure 5a,b, respectively. Catalase from strain I1P was active in a wide range of temperatures (from 20 to 70 °C), showing optimal activity at 50 °C (at pH 7.0) (Figure 5a). The optimal pH for enzyme activity was 7.0 (at 50 °C) (Figure 5b). Although it is not clear why an apparent two pH optimum were observed in this purification, the optimal pH (pH 7.0) was always the same between different batches of purifications. Surprisingly, the optimal temperature for enzyme activity of this CAT was found to be higher than the upper temperature at which strain I1P was able to grow (37 °C), indicating that this enzyme is thermoactive and more similar to thermophilic than psychrophilic homologs. It is important to mention that although the temperature range for enzyme activity reported here was between 20 and 70 °C, this catalase was even active at 0 °C, as shown by the production of bubbles due to the enzymatic decomposition of H_2_O_2_ in the presence of catalase (Supplementary Figure S3). Quantitative data could not be obtained below 20 °C due to experimental constraints, thus not allowing spectrophotometric enzymatic assays to be performed with temperature control at lower temperatures.

**Figure 6:**
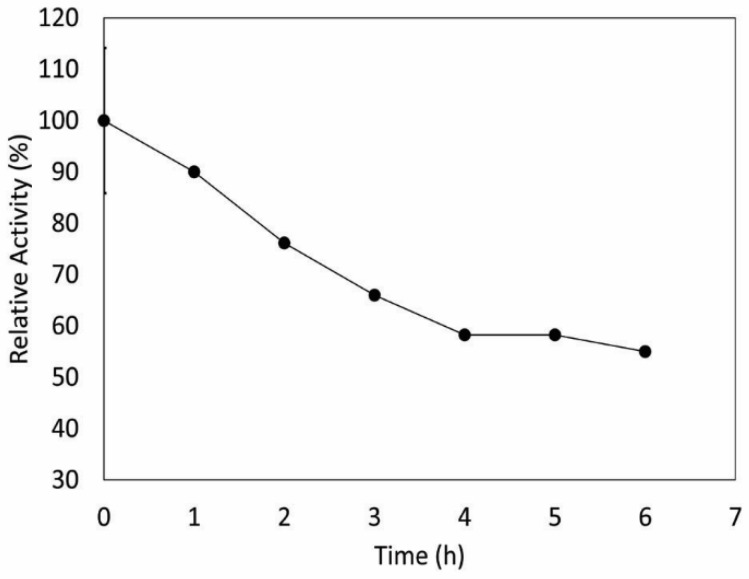
Thermostability of I1P catalase at 50 °C. Measurements were performed at pH 7.0 and 50 °C. Here, 100% of catalase activity represents the maximum activity measured. Measurements were performed in triplicate.

To further evaluate the thermoactive properties of I1P CAT, the themostability of this enzyme was studied. Figure 6 shows that the enzyme retained around 60% of its activity after 6 h of incubation at 50 °C. In comparison to other catalases isolated from psychrophiles or facultative psychrophiles, I1P CAT was more thermostable than the enzymes from *Vibrio rumoiensis* S-1T or the Antarctic bacterium *Bacillus* sp. strain N2a, whose catalases only retained 70% of its activity after 15 min of incubation at 50 °C [40] and 40% of its activity after 40 min of incubation at 50 °C [57], respectively. These results further support the observation that CAT from strain I1P is thermoactive, an important property in the biotechnological industry [58].

Although it has been previously reported that other catalases from psychrophilic microorganisms could share some characteristics (i.e., themostability, optimal temperature) with their mesophilic homologues [57], to our knowledge, this is the first report of a CAT from a psychrotolerant microorganism that is more similar to thermophilic homologs. Although the structural mechanisms determining these thermal characteristics are not known, it has been previously suggested that stabilizing interactions of the secondary, tertiary, and quaternary structure throughout the protein are likely involved in conferring these properties [59]. Further studies are required to investigate the structural characteristics of this thermoactive enzyme responsible for its thermostability and activity at thermophilic temperatures. 

Another remarkable feature of the CAT enzyme from strain I1P was its high stability at room temperature and at freezing conditions. I1P CAT retains 74% of its activity for 30 days at room temperature, 66% of its activity for 137 days at 4 °C, and 100% of its activity after 137 days at −20 °C (Supplementary Figure S4). Furthermore, this enzyme resists freezing and thawing cycles, retaining 66% of its activity after 33 of these cycles. These characteristics, in addition to the ability of the enzyme to be active in a wide range of temperatures, being thermoactive and thermostable, make this enzyme a good candidate for industrial applications. Moreover, the catalytic activity of the CAT enzyme from strain I1P (Table 3) was shown to be higher than the activity of several commercial catalases currently sold in the market (Table 4), reaching 7447 U/mg in its last purification step.

To further characterize this CAT enzyme, the effect of different inhibitors, including 3-amino-1,2,4 triazole (75 mM), potassium cyanide (10 mM), and H_2_O_2_ (80 mM), on enzyme activity was also studied. The catalytic activity of the purified CAT from strain I1P was inhibited by 70% by 3-amino-1,2,4-triazole and 90% by potassium cyanide. Treatment with H_2_O_2_ only inhibited 30% of the enzyme activity. On the other hand, organic solvents, like ethanol and chloroform, did not have a strong effect on CAT activity, since the enzyme was able to maintain 50% of its activity after 41 h of exposure to these organic solvents. The inhibition pattern of I1P CAT activity, specifically its sensitivity to 3-amino-1,2,4-triazole and its stability against chemical denaturation by organic solvents, suggests that CAT from strain I1P belongs to the monofunctional catalase group. This is consistent with the homotetrameric nature of the I1P CAT, which is a characteristic of most monofunctional catalases [40,60,61]. In contrast, catalase-peroxidases are more sensitive to heat, organic solvents, and H_2_O_2_ than the monofunctional catalases and are insensitive to 3-amino-1,2,4-triazole [40]. The sensibility to potassium cyanide, a typical inhibitor of heme proteins, suggests that the subunits of I1P CAT possess a ferric heme, a shared characteristic between monofunctional catalases and catalase-peroxidases but absent in the third class of catalases, known as manganese-catalases [62]. The observation that the CAT enzyme from strain I1P belongs to the monofunctional catalase group was supported by enzyme activity analysis that showed an absence of peroxidase activity in the purified enzyme.

## 4. Conclusions

The evidence presented here indicates that *Serratia* sp. strain I1P is resistant to UV-C radiation and is well-adapted to cold temperatures. These physiological adaptations are likely consequences of the Antarctic environmental pressures (i.e., high UV radiation, cold temperatures), which select for microorganisms that can withstand these extreme conditions in order to thrive. Among the properties that allow strain I1P to deal with ROS generation caused by the environmental conditions present in Antarctica, we suggest that CAT plays a key role in the antioxidant defense mechanisms of this bacterium. The characterization of the purified CAT from strain I1P showed that this enzyme was thermoactive with a remarkable thermostability, considering that this enzyme was purified from a psychrotolerant microorganism. Moreover, in addition to its thermal stability and activity at thermophilic temperatures, this enzyme was shown to be stable for a long time at room temperature and during freezing conditions. These stability properties, in conjunction with its good catalytic activity, make this CAT enzyme a good candidate for industrial applications. These observations, in addition to previous investigations reporting the isolation of novel enzymes with potential biotechnological applications from Antarctic microorganisms [1,16], underscore the importance of Antarctica as an interesting source of new extreme microorganisms containing stable and active enzymes.

## Figures and Tables

**Table 1:** Morphological, physiological, and biochemical characterization of strain I1P.

Characteristics	I1P
Morphology	rod
Gram stain	negative
Temperature range (optimum)	4–37 °C (22 °C)
pH range (optimum)	5–10 (7–9)
Salinity range (optimum)	2–19% (2–6%)
API 20 E tests	
β-galactosidase	−
Arginine dihydrolase	−
Lysine decarboxylase	−
Ornithine decarboxylas	−
Citrate utilization	−
H_2_S production	−
Urease	−
l-tryptophane deaminase	+
Indol production	−
Acetoin production	−
Gelatinase	+
d-glucose (fermentation-oxidation)	−
d-mannitol (fermentation-oxidation)	−
Inositol (fermentation-oxidation)	−
d-sorbitol (fermentation-oxidation)	−
l-rhamnose (fermentation-oxidation)	−
d-sucrose (fermentation-oxidation)	−
d-melibiose (fermentation-oxidation)	−
Amygdalin (fermentation-oxidation)	−
l-arabinose (fermentation-oxidation)	−
Nitrate reduction to NO_2_	+
Nitrate reduction to N_2_	+

+ positive reaction; − negative reaction.

**Table 2:** Viability of cells exposed to UV-C radiation.

	*E. coli*		GEW1		I1P	
Time (min)	Viability *	UV Dose ^#^	Viability *	UV Dose ^#^	Viability *	UV Dose ^#^
0	100	0	100	0	100	0
5	0	0.19	4.5	0.19	75.0	0.19
30	0	1.26	0.08	1.26	18.5	1.26
60	0	2.63	0.02	2.63	5.0	2.63
120	0	6.33	0.01	6.33	2.0	6.33

* Viability in %, 100% of viable cells represents the maximum number of colonies counted. Measurements were performed in quintuplicate. ^#^ UV dose in Jls/cm^2^.

**Table 3:** Purification of catalase from I1P.

Purification Step	Total Protein (mg)	Total Activity (U)	Specific Activity (U/mg)	Purification (Fold)
Crude Extract	2320	59,143	25	1
DEAE- Sepharose FF	26	82,141	318	13
Superdex-200	13	81,321	6265	251
Q-HiTrap FF	2	12,511	7447	298

**Table 4:** Commercially available catalases and their uses.

Manufacturer	Commercial Name	Source	Applications	Specific Activity (U/mg)
Novozyme	Terminox Ultra	No data	Textile industry	10
	Catazyme	*Aspergillus niger* *	Food and textile industry	4960
Sigma-Aldrich	Catalase	*A. niger*	Research market	>4000
	Catalase	*Corynebacterium glutamicum*		>71,428
	Catalase	*Micrococcus luteus*		65–150
Biocatalysts	Catalase 929 L	*A. niger*	Food industry	16.5
Calzyme	Catalase	*A. niger*	No data	2000
This study	Catalase	*Serratia* sp. I1P	Research market	7447

* Recombinant version.

## Supplementary Materials

The following are available online at https://www.mdpi.com/2076-2607/8/1/95/s1, Figure S1: Optical micrograph of strain I1P, Figure S2: Molecular mass determination of I1P catalase subunits and native enzyme, Figure S3: Qualitative assay to detect catalase activity at 0 °C, Figure S4: Stability of I1P catalase, Table S1: Viability of *E. coli* BL21 cells exposed to UV-C radiation.

## References

[1] Flores P.A., Amenábar M.J., Blamey J.M., Satyanarayana T., Littlechild J., Kawarabayasi Y. (2013). Hot Environments from Antarctica. Source of Thermophiles and Hyperthermophiles, with Potential Biotechnological Applications. Thermophilic Microbes in Environmental and Industrial Biotechnology.

[2] Smith R.C., Prézelin B.B., Baker K.S., Bidigare R.R., Boucher N., Coley T.R., Karentz D., MacIntyre S., Matlick H.A., Menzies D. (1992). Ozone depletion: Ultraviolet radiation and phytoplankton biology in Antarctic waters. Science.

[3] Correa-Llantén D.N., Amenábar M.J., Blamey J.M. (2012). Antioxidant capacity of novel pigments from an Antarctic bacterium. J. Microbiol..

[4] Amenabar M.J., Flores P.A., Pugin B., Boehmwald F.A., Blamey J.M. (2013). Archaeal diversity from hydrothermal systems of deception island, Antarctica. Polar Biol..

[5] Sies H. (1997). Oxidative stress: Oxidants and antioxidants. Exp. Physiol. Trans. Integr..

[6] Briviba K., Klotz L.O., Sies H. (1997). Toxic and signaling effects of photochemically or chemically generated singlet oxygen in biological systems. Biol. Chem..

[7] Wu H., Gao K., Villafañe V.E., Watanabe T., Helbling E.W. (2005). Effects of solar UV radiation on morphology and photosynthesis of filamentous cyanobacterium *Arthrospira platensis*. Appl. Environ. Microbiol..

[8] Imlay J.A. (2003). Pathways of oxidative damage. Annu. Rev. Microbiol..

[9] Correa-Llantén D.N., Amenábar M.J., Muñoz P.A., Monsalves M.T., Castro M.E., Blamey J.M. (2014). *Alicyclobacillus* sp. strain CC2, a thermo-acidophilic bacterium isolated from deception island (Antarctica) containing a thermostable superoxide dismutase enzyme. Adv. Polar Sci..

[10] Mondino L.J., Asao M., Madigan M.T. (2009). Cold-active halophilic bacteria from the ice-sealed Lake Vida, Antarctica. Arch. Microbiol..

[11] Cabiscol E., Tamarit J., Ros J. (2000). Oxidative stress in bacteria and protein damage by reactive oxygen species. Int. Microbiol..

[12] Correa-Llantén D.N., Amenabar M.J., Blamey J.M. (2012). Resistance to hypoosmotic shock of liposomes containing novel pigments from an Antarctic bacterium. Microbiol. Biotechnol. Lett..

[13] Dieser M., Greenwood M., Foreman C.M. (2010). Carotenoid Pigmentation in Antarctic Heterotrophic Bacteria as a Strategy to Withstand Environmental Stresses. Arct. Antarct. Alp. Res..

[14] Monsalves M.T., Amenabar M.J., Ollivet-Besson G.P., Blamey J.M. (2013). Effect of UV radiation on a thermostable superoxide dismutase purified from a thermophilic bacterium isolated from a sterilization drying oven. Protein Pept. Lett..

[15] Halliwell B. (2006). Reactive species and antioxidants. Redox biology is a fundamental theme of aerobic life. Plant Physiol..

[16] Boehmwald F., Muñoz P., Flores P., Blamey J.M., Rampelotto P. (2016). Functional screening for the discovery of new extremophilic enzymes. Biotechnology of Extremophiles: Grand Challenges in Biology and Biotechnology.

[17] Zheng Z., Jiang Y., Miao J., Wang Q.F., Zhang B.T., Li G.Y. (2006). Purification and characterization of a cold-active iron superoxide dismutase from a psychrophilic bacterium, *Marinomonas sp*. NJ522. Biotechnol. Lett..

[18] Ji M., Barnwell C., Grunden A. (2015). Characterization of recombinant glutathione reductase from the psychrophilic Antarctic bacterium *Colwellia psychrerythraea*. Extremophiles.

[19] Kaushal J., Mehandia S., Singh G., Raina A., Arya S.K. (2018). Catalase enzyme: Application in bioremediation and food industry. Biocatal. Agric. Biotechnol..

[20] Liu X., Kokare C., Brahmachari G., Demain A.L., Adrio J.L. (2017). Microbial Enzymes of Use in Industry. Biotechnology of Microbial Enzymes.

[21] Lončar N., Fraaije M.W. (2015). Catalases as biocatalysts in technical applications: Current state and perspectives. Appl. Microbiol. Biotechnol..

[22] Fruhwirth G.O., Paar A., Gudelj M., Cavaco-Paulo A., Robra K.H., Gubitz G.M. (2002). An immobilized catalase peroxidase from the alkalothermophilic *Bacillus* SF for the treatment of textile bleaching effluents. Appl. Microbiol. Biotechnol..

[23] Yu Z., Zheng H., Zhao X., Li S., Xu J., Song H. (2016). High level extracellular production of a recombinant alkaline catalase in *E. coli BL21* under ethanol stress and its application in hydrogen peroxide removal after cotton fabrics bleaching. Bioresour. Technol..

[24] Gudelj M., Fruhwirth G.O., Paar A., Lottspeich F., Robra K., Cavaco-Paulo A., Gübitz G.P. (2001). A catalase-peroxidase from a newly isolated thermoalkaliphilic *Bacillus sp*. with potential for the treatment of textile bleaching effluents. Extremophiles.

[25] Amenabar M.J., Colman D.R., Poudel S., Roden E.E., Boyd E.S. (2018). Electron acceptor availability alters carbon and energy metabolism in a thermoacidophile. Environ. Microbiol..

[26] Amenabar M.J., Boyd E.S. (2018). Mechanisms of mineral substrate acquisition in a thermoacidophile. Appl. Environ. Microbiol..

[27] Boyd E.S., Cummings D.E., Geesey G.G. (2007). Mineralogy influences structure and diversity of bacterial communities associated with geological substrata in a pristine aquifer. Microb. Ecol..

[28] Hall T.A. (1999). BioEdit: A user-friendly biological sequence alignment editor and analysis program for Windows 95/98/NT. Nucleic Acid Symp. Ser..

[29] Altschul S., Madden T., Schaffer A., Zhang J., Zhang Z., Miller W., Lipman D. (1997). Gapped BLAST and PSI-BLAST: A new generation of protein database search programs. Nucleic Acid Res..

[30] Tamura K., Stecher G., Peterson D., Filipski A., Kumar S. (2013). MEGA6: Molecular Evolutionary Genetics Analysis version 6.0. Mol. Biol. Evol..

[31] Saitou N., Nei M. (1987). The neighbor-joining method: A new method for reconstructing phylogenetic trees. Mol. Biol. Evol..

[32] Tamura K., Nei M., Kumar S. (2004). Prospects for inferring very large phylogenies by using the neighbor-joining method. Proc. Natl. Acad. Sci. USA.

[33] Correa-Llantén D., Larraín-Linton J., Muñoz P.A., Castro M., Boehmwald F., Blamey J.M. (2013). Characterization of the thermophilic bacterium *Geobacillus* sp. strain GWE1 isolated from a sterilization oven. Microbiol. Biotechnol. Lett..

[34] Beers R.F., Sizer I.W. (1952). A spectrophotometric method for measuring the breakdown of hydrogen peroxide by catalase. J. Biol. Chem..

[35] Chance B., Maehly A.C., Glick D. (1955). Assay of Catalases and Peroxidases. Methods of Biochemical Analysis.

[36] Bradford M.M. (1976). A rapid and sensitive method for the quantitation of microgram quantities of protein utilizing the principle of protein-dye binding. Anal. Biochem..

[37] Amenabar M.J., Blamey J.M. (2012). Purification and characterization of a thermostable glutamate dehydrogenase from a thermophilic bacterium isolated from a sterilization drying oven. Biochem. Mol. Biol..

[38] Laemmli U.K. (1970). Cleavage of structural proteins during the assembly of the head of Bacteriophage T4. Nature.

[39] Allgood G.S., Perry J.J. (1986). Characterization of a manganese-containing catalase from the obligate thermophile *Thermoleophilum album*. J. Bacteriol..

[40] Yumoto I., Ichihashi D., Iwata H., Istokovics A., Ichise N., Matsuyama H., Okuyama H., Kawasaki K. (2000). Purification and characterization of a catalase from the facultatively psychrophilic bacterium *Vibrio rumoiensis* S-1T exhibiting high catalase activity. J. Bacteriol..

[41] Kang Y.S., Lee D.H., Yoon B.J., Oh D.C. (2006). Purification and characterization of a catalase from photosynthetic bacterium *Rhodospirillum rubrum* S1 grown under anaerobic conditions. J. Microbiol..

[42] Chattopadhyay M., Raghu G., Sharma Y., Biju A., Rajasekharan M., Shivaji S. (2011). Increase in oxidative stress at low temperature in an Antarctic bacterium. Curr. Microbiol..

[43] Stackebrandt E., Ebers J. (2006). Taxonomic parameters revisited: Tarnished gold standards. Microbiol. Today.

[44] Janda J.M., Abbott S.L. (2007). 16S rRNA gene sequencing for bacterial identification in the diagnostic laboratory: Pluses, perils, and pitfalls. J. Clin. Microbiol..

[45] Grimont P.A., Grimont F., Starr M.P. (1978). *Serratia proteamaculans* (Paine and Stansfield) comb. nov., a senior subjective synonym of *Serratia liquefaciens* (Grimes and Hennerty) Bascomb et al. Int. J. Syst. Evol. Microbiol..

[46] Amara U. (2015). Molecular and Biochemical Characaterization of Plant Growth Promoting Rhizobacteria for Enhancing Crop Yield. Ph.D. Thesis.

[47] Grimont F., Grimont P.A., Dworkin M., Falkow S., Rosenberg E., Schleifer K.H., Stackebrandt E. (2006). The Genus *Serratia*. The Prokaryotes.

[48] Sánchez L.A., Gómez F.F., Delgado O.D. (2009). Cold-adapted microorganisms as a source of new antimicrobials. Extremophiles.

[49] Shivaji S., Reddy G., Aduri R., Kutty R., Ravenschlag K. (2004). Bacterial diversity of a soil sample from Schirmacher Oasis, Antarctica. Cell. Mol. Biol..

[50] Xiao X., Li M., You Z., Wang F. (2007). Bacterial communities inside and in the vicinity of the Chinese Great Wall Station, King George Island, Antarctica. Antarct. Sci..

[51] Santos A.L., Gomes N.C., Henriques I., Almeida A., Correia A., Cunha Â. (2012). Contribution of reactive oxygen species to UV-B-induced damage in bacteria. J. Photochem. Photobiol. B.

[52] Krisko A., Radman M. (2010). Protein damage and death by radiation in *Escherichia coli* and *Deinococcus radiodurans*. Proc. Natl. Acad. Sci. USA.

[53] Salma K.B., Lobna M., Sana K., Kalthoum C., Imene O., Abdelwaheb C. (2016). Antioxidant enzymes expression in *Pseudomonas aeruginosa* exposed to UV-C radiation. J. Basic Microbiol..

[54] Meng J.Y., Zhang C.Y., Fen Z., Wang X.P., Lei C.L. (2009). Ultraviolet light-induced oxidative stress: Effects on antioxidant response of *Helicoverpa armigera* adults. J. Insect Physiol..

[55] Arenas F.A., Díaz W.A., Leal C.A., Pérez-Donoso J.M., Imlay J.A., Vásquez C.C. (2010). The *Escherichia coli* btuE gene, encodes a glutathione peroxidase that is induced under oxidative stress conditions. Biochem. Biophys. Res. Commun..

[56] Alquéres S., Meneses C., Rouws L., Rothballer M., Baldani I., Schmid M., Hartmann A. (2013). The bacterial superoxide dismutase and glutathione reductase are crucial for endophytic colonization of rice roots by *Gluconacetobacter diazotrophicus* PAL5. Mol. Plant Microbe Interact..

[57] Wang W., Sun M., Liu W., Zhang B. (2008). Purification and characterization of a psychrophilic catalase from antarctic bacillus. Can. J. Microbiol..

[58] Danson M.J., Hough D.W., Russell R.J., Taylor G.L., Pearl L. (1996). Enzyme thermostability and thermoactivity. Protein Eng..

[59] Switala J., Loewen P.C. (2002). Diversity of properties among catalases. Arch. Biochem. Biophys..

[60] Lorentzen M.S., Moe E., Jouve H.M., Willassen N.P. (2006). Cold adapted features of *Vibrio salmonicida* catalase: Characterisation and comparison to the mesophilic counterpart from *Proteus mirabilis*. Extremophiles.

[61] Díaz A., Loewen P.C., Fita I., Carpena X. (2012). Thirty years of heme catalases structural biology. Arch. Biochem. Biophys..

[62] Chelikani P., Fita I., Loewen P.C. (2004). Diversity of structures and properties among catalases. Cell. Mol. Life Sci..

