# Supplementary material for: Isolation of a Psychrotolerant and UV-C-Resistant Bacterium from Elephant Island, Antarctica with a Highly Thermoactive and Thermostable Catalase
Source: Microorganisms. 2020 Jan 10;8(1):95. doi: 10.3390/microorganisms8010095 (PMC7022778; doi:10.3390/microorganisms8010095)
Supplement: Supplementary file 1 [file microorganisms-08-00095-s001.pdf]

1. Supplementary Figures and Tables

Supplementary Table 1. Viability of *E. coli* BL21 cells exposed to UV-C radiation.

	E. coli BL21		*E. coli* BL21 (recombinant)	
Time (min.)	Viability*	UV dose#	Viability*	UV dose#
0	100	0	100	0
5	0	0.19	51.2	0.19
35	0	1.26	40.5	1.26

*Viability in %, 100% of viable cells represents the maximum number of colonies counted. Measurements were performed in triplicate. # UV dose in Jls/cm^2.

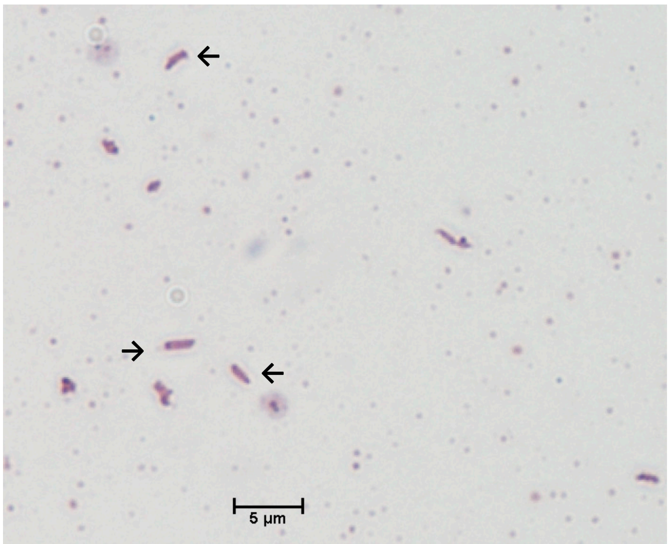

Supplementary Figure 1. Optical micrograph of strain I1P. Cells were stained using the gram procedure. Arrow indicate rod shape microorganisms. See main text for more details.

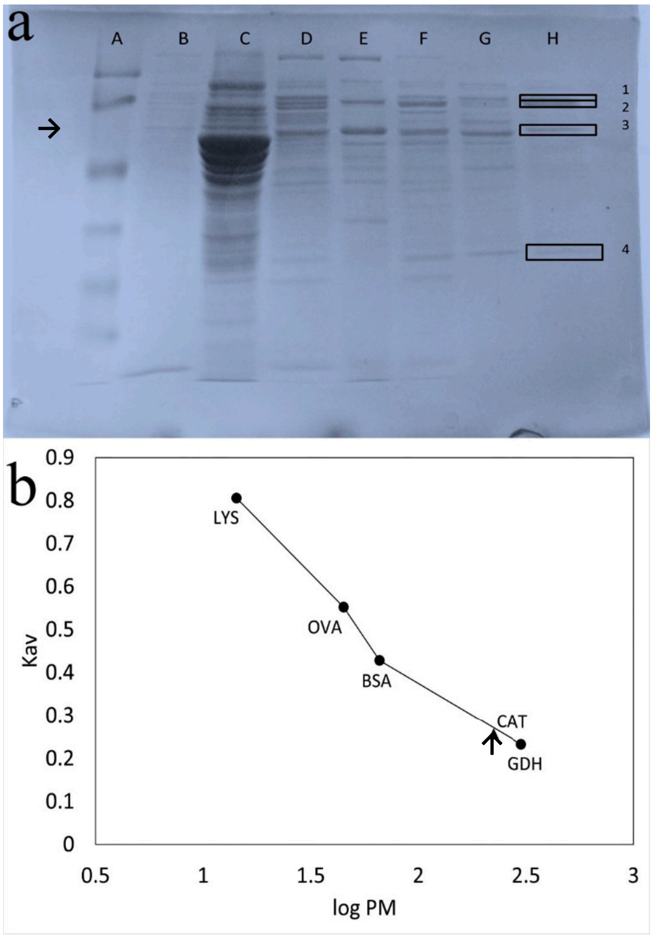

Supplementary Figure 2. Molecular mass determination of I1P catalase subunits and native enzyme by SDS-PAGE (a) and size exclusion liquid chromatography (b) respectively. Panel (a) shows the result of protein purification of I1P catalase. Four predominant bands were observed in the last purification step (lane H). These bands where identified by MALDI TOF/TOF indicating that only band 3 corresponded to a catalase subunit. Lane A: molecular weight marker (20-120 kDa); Lane B: catalase enzyme from sigma (positive control); Lane C: crude extract; Lane D: DEAE fraction; Lanes E and F: Superdex fractions; Lanes G and H: Q-HiTrap fractions. Arrow denotes the position of catalase subunit. Panel (b) shows the calibration curve used to calculate the apparent molecular mass of the native catalase. Partition coefficient (Kav) of I1P catalase (0.256) is indicated by an arrow. Lys: lysozyme; OVA: ovalbumin; BSA: bovine serum albumin; GDH: glutamate dehydrogenase.

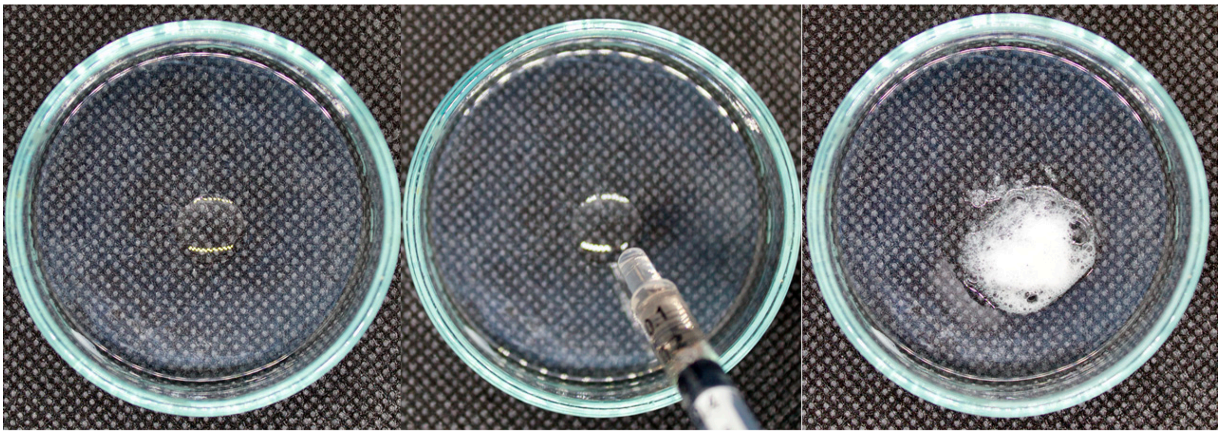

Supplementary Figure 3. Qualitative assay to detect catalase activity at 0°C. Left panel shows a drop of H_2O_2 used as a substrate for catalase activity. Middle panel shows the addition of the catalase enzyme. Right panel shows the development of bubbles after the addition of catalase due to the enzymatic decomposition of H_2O_2 to H_2O and O_2.

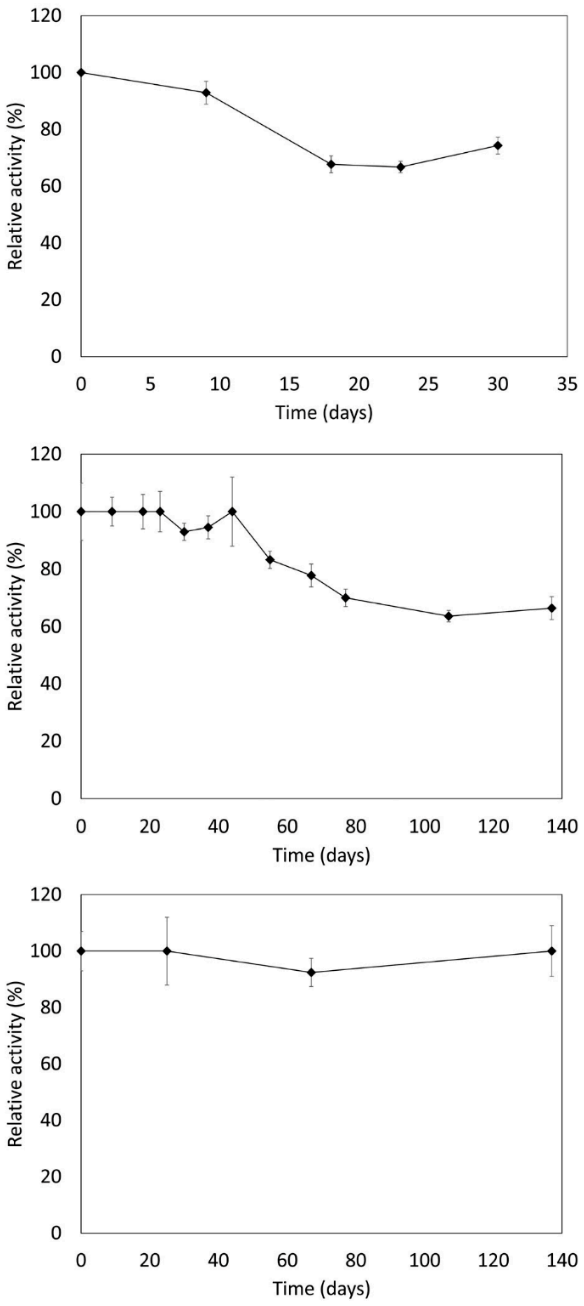

Supplementary Figure 4. Stability of I1P catalase at room temperature (top panel), 4°C (middle panel) and -20°C (bottom panel). Measurements were performed in triplicate.
